# Microgravity as a translational model for metabolic dysfunction: implications for obesity, insulin resistance, steatotic liver and pancreatic diseases

**DOI:** 10.3389/fendo.2026.1820707

**Published:** 2026-04-24

**Authors:** Mathew Vadukoot Lazar, Arun C. S. Menon, Rajesh Gopalakrishna

**Affiliations:** 1 Department of Gastroenterology, Lifecare Hospital, Abu Dhabi, United Arab Emirates; 2 Department of Endocrinology, Aster Clinic, Bur Dubai (AJMC), Dubai, United Arab Emirates; 3 Department of Gastroenterology, Apollo Adlux Hospital, Angamaly, Kerala, India

**Keywords:** ectopic fat, insulin resistance, metabolic syndrome, microgravity, sedentary lifestyle, steatotic liver and pancreatic diseases

## Abstract

**Purpose:**

The long-term effects of physical inactivity, whether due to microgravity in spaceflight or an inactive lifestyle on Earth, can lead to a range of metabolic problems. This review examines the physiological response to reduced gravity and contemporary inactivity, with a focus on obesity, insulin resistance, type 2 diabetes, and hepatic and pancreatic steatosis, all of which are associated with metabolic dysfunction (MASLD, MASPD).

**Methods:**

A narrative review approach was used, integrating evidence from spaceflight research, bed rest studies, and clinical trial data to examine the metabolic consequences of reduced mechanical loading and physical inactivity.

**Results:**

Available evidence suggests that mechanical load deprivation alters fat distribution, promotes visceral adiposity, and initiates inflammation and immune-metabolic reprogramming. Emerging countermeasures, such as vibration platforms, resistive suits, and omics-directed personalized therapies, have been explored as potential strategies to mitigate these changes. A 12-week cascade model is proposed to illustrate the disease progression of metabolic dysfunction under reduced mechanical loading.

**Conclusion:**

This review has brought together aerospace physiology and metabolic medicine, highlighting microgravity as a translational model that helps improve our understanding and mitigate lifestyle-induced metabolic disorders on Earth.

**Methods:**

A comprehensive literature search was performed using electronic databases, including PubMed/MEDLINE, Scopus, and Web of Science. The search strategy incorporated a combination of the following keywords: Microgravity, Sedentary lifestyle, Insulin resistance, Ectopic fat, Metabolic syndrome, Steatotic Liver, and Pancreatic Diseases. Studies were selected based on relevance to the topic and included human studies (astronaut data, clinical cohorts, and bed rest trials), animal models of simulated microgravity, and *in vitro* mechanical studies. Articles not directly related to metabolic and endocrine outcomes of mechanical unloading were excluded. Given the heterogeneity of study designs and outcomes, findings were synthesized using a structured narrative approach.

## Introduction:

Microgravity is the near-weightlessness of spaceflight, which significantly reduces the force of gravity acting on the body (1). Such an environment changes the biomechanical loading, particularly damaging the musculoskeletal and metabolic systems (2). Ground-based models can mimic microgravity-like conditions, including head-down tilt bed rest and dry immersion; both are widely used to investigate physiological deconditioning in the absence of gravitational stress (3). The models are becoming useful for investigating the long-term biological effects of sedentary behavior, a significant public health issue in contemporary Societies (4, 5). The physiological alterations associated with sedentary lifestyles, characterized by minimal mechanical loading, are similar to those observed in microgravity, including muscle atrophy, visceral fat accumulation, and metabolic dysregulation (6, 7). This overlap underscores the utility of microgravity as a translational model for exploring mechanisms underlying lifestyle-related metabolic diseases.

Investigating the physiological artifacts of gravitational unloading provides mechanistic insight into the role of inactivity in disease pathogenesis (8). Controlled microgravity and bed rest studies allow for precise assessment of how reduced mechanical forces disrupt metabolic homeostasis, energy balance, and immune-inflammatory processes (3, 9). Notably, physical inactivity and gravitational deprivation have become independent risk factors for the development of obesity, insulin resistance, and ectopic lipid deposition in hepatic and pancreatic tissues (3, 10).

The review synthesizes current evidence on the metabolic effects of altered gravity, focusing on obesity, Type 2 diabetes, and steatotic liver and pancreatic diseases related to metabolic dysfunction (MASLD and MASPD). The comparative physiological effects of Earth gravity and microgravity are analyzed, and new therapeutic approaches that mimic gravitational loading are considered. Additionally, the review examines how space mission omics data can inform individual exercise and prevention programs. Finally, the development of metabolic dysfunction due to decreased mechanical loading is described using a temporal cascade model. Accordingly, this review proposes mechanical unloading as an emerging endocrine -metabolic mechanism linking physical inactivity to insulin resistance and ectopic fat deposition.

## Microgravity as a model for sedentary lifestyle

A prolonged period of microgravity, as in spaceflight, leads to rapid deterioration of various physiological systems, particularly the musculoskeletal, cardiovascular, and metabolic systems (Human studies: astronaut data) (11). Significant losses in skeletal muscle mass, bone mineral density, and insulin sensitivity have been observed even during short-term missions (Human studies) (12). These physiological adaptations are mainly due to the absence of Earth’s gravity, an essential mechanical stimulus for maintaining structural and metabolic homeostasis (13).

It has been established that, within a few days to weeks, bed-rest study participants exhibit significant losses in muscle cross-sectional area, basal metabolic rate, and insulin sensitivity, which are virtually identical to the initial metabolic dysfunction observed in spaceflight (Human studies: Bed-rest trials) (14). Such ground-based models are invaluable for studying the pathophysiology of disuse and inactivity because they minimize confounding factors such as nutrition and environmental variation (15).

The biological effects of microgravity are strikingly similar to those of contemporary sedentary lifestyles (Human observational Data) (16). Sedentary behavior, defined as low energy expenditure while sitting or reclining, has been linked to increased visceral adiposity, chronic inflammation, and a predisposition to metabolic syndrome (17). A recent study conducted in the slum regions of Karachi found that 42% of individuals living with type 2 diabetes exhibited sedentary behavior, with prevalence notably higher among those with obesity (22%) and individuals from low socioeconomic backgrounds (61%) (18). The above similarities highlight the significance of microgravity as a translational model for studying immobility-induced metabolic dysfunction. **Table 1** summarizes key physiological similarities between microgravity and sedentary conditions, underscoring their relevance as research analogs.

**Table 1:** Microgravity vs. sedentary lifestyle – a translational model of physiological deterioration.

Physiological impact	Microgravity (Spaceflight/Bed Rest)	Sedentary lifestyle (Earth)	Key references
Muscle Loss	Rapid atrophy, ↓ muscle cross-sectional area	Progressive sarcopenia, ↓ activity-related muscle tone	(12, 14) (Human studies)
Bone Density Loss	↓ Bone mineral density due to unloading	↓ Bone strength from inactivity	(13) (Human studies)
Insulin Sensitivity	Decreased within days; early insulin resistance	Associated with low energy expenditure and fat gain	(15, 17) (Human, Animal studies)
Visceral Adiposity	Redistribution to VAT without total weight gain	Central obesity is common in sedentary adults	(16) (Human, Animal studies)
Inflammation	↑ TNF-α and IL-6 levels due to visceral fat accumulation	Chronic low-grade inflammation observed	(11, 18) (Human, Animal studies)
Cardiovascular Deconditioning	↓ Baroreflex sensitivity and vascular tone	Poor cardiovascular fitness, ↑ risk of hypertension	(12, 18) (Human studies)

## Metabolic disorders triggered by microgravity/sedentary conditions

The absence of gravitational loading and reduced physical activity initiates metabolic disturbances, including obesity, insulin resistance (81%), ectopic fat accumulation, including the pancreas and liver (50%) (Human studies: clinical and epidemiological data) (19, 20).

A lack of gravitational loading, along with reduced physical activity, leads to metabolic imbalances, such as obesity, insulin resistance, and ectopic fat deposition. It is noteworthy that 50% teenagers with concurrent obesity and non-alcoholic fatty liver disease were found to have pancreatic lipid deposition, and 81% of them developed insulin resistance.(Human studies) (21, 22). Gamma-glutamyl transferase (GGT) was a good predictor of fatty pancreas in adult populations, with an odds ratio of 7.33(Human studies). Experimental models further reinforce these clinical findings(Animal data), which demonstrate that disuse conditions promote visceral adiposity and systemic inflammation, key contributors to ectopic fat deposition and metabolic dysfunction (23, 24).

Diabetes type 2 is increasingly recognized as a potential consequence of prolonged mechanical unloading and physical inactivity. Skeletal muscle displays impaired glucose uptake in the insulin-stimulated state within days of inactivity (Human studies: Bed-rest Trials) (25, 26). The observed effect persists even in the absence of weight gain, suggesting a perturbation of mechanical signaling pathways vital to glycemic regulation. At the same time, physical inactivity lowers fatty acid oxidation and increases hepatic lipogenesis. These changes enable the development of steatotic liver and pancreatic disease (associated with metabolic dysfunction) (MASLD), (MASPD), even in the case of people who remain at constant levels of caloric intake (24, 27).

New findings implicate reduced loading in pancreatic steatosis and beta-cell dysfunction, which are characteristics of metabolic dysfunction in steatotic pancreas disease (MASPD) (28). Taken together, these explain the contributions of gravitational forces and mechanical strain to the physiological modulation of metabolic processes (29). The depletion initiates a predictable, quantifiable destabilization of metabolic homeostasis, making microgravity an attractive model for understanding lifestyle-induced metabolic disease. These observations reinforce the clinical relevance of mechanical loading as a modifiable factor in metabolic risk. **Table 2** provides a consolidated overview of the key metabolic disorders associated with mechanical unloading, outlining triggers, clinical data, and supporting literature.

**Table 2:** Summary of metabolic disorders associated with mechanical unloading, detailing underlying mechanisms, clinical prevalence, and key supporting studies.

Disorder	Mechanism/Trigger	Evidence/Prevalence	Key studies
Obesity (Visceral)	Reduced energy expenditure; disuse promotes adiposity	Visceral fat gain without weight increase	(23) (Human, Animal studies)
Insulin Resistance	Impaired glucose uptake in unloaded muscle	81% of obese adolescents with NAFLD had insulin resistance	(22, 26) (Human, Animal studies)
MASLD (Liver Steatosis)	Increased lipogenesis; reduced fatty acid oxidation	Develops even without caloric excess	(24, 27) (Human studies)
MASPD (Pancreatic Steatosis)	Lipid infiltration; beta-cell dysfunction	50% of adolescents with obesity & NAFLD; linked to insulin resistance	(21, 28) (Human, Animal studies)
Fatty Pancreas (Adults)	Marker of metabolic dysfunction	GGT ↑ (OR = 7.33) for detecting fatty pancreas	(19) (Human studies)
Systemic Inflammation	Visceral adipose cytokine release (e.g., TNF-α, IL-6)	Promotes insulin resistance and ectopic fat storage	(23, 29) (Human, *in-vitro* studies)

## Comparative effects of normal gravity vs. microgravity

Under Earth’s gravity, the human body is continuously subjected to mechanical forces that are essential for maintaining the structural and functional integrity of various organ complexes (30). The gravitational force on Earth keeps skeletal muscle in tone, maintains bone mineral density, regulates the heart and cardiovascular system, and ensures metabolic homeostasis. On the other hand, the lack of mechanical loading in microgravity, as in orbital missions, induces rapid physiological deconditioning (31, 32).

It is essential to note that long-term extraterrestrial missions are always associated with significant losses in skeletal muscle mass, bone mineral density, and cardiovascular function in astronauts (33). Losses in muscle strength and bone resorption increase even in the case of a short-term flight (even 5–16 days). The primary cause of these early changes is the lack of gravitational loading, which deprives the musculoskeletal system of the mechanical stimuli required to maintain musculoskeletal integrity (34).

Astronauts experience accelerated metabolic changes, including reduced fat breakdown and insulin resistance. Similar effects can be measured in ground-based analogs, such as head-down tilt bed rest, indicating the repeatability of these impacts in non-spaceflight conditions (22). All these observations together highlight the crucial role of gravity as a stabilizing and protective force. Its sustained presence coordinates musculoskeletal and systemic metabolic control, whereas its absence initiates rapid disruption of homeostasis, underscoring the urgency of countermeasures in clinical practice and spaceflight operations. **Table 3** outlines the key physiological differences between standard gravity and microgravity or bedrest environments.

**Table 3:** Key physiological changes in normal gravity vs. microgravity.

Parameter	Normal gravity	Microgravity/Bedrest
Muscle Mass	Maintained via load-bearing activity	Rapid atrophy, strength loss within days
Bone Density	Preserved through mechanical loading	Increased resorption, density loss
Fat Metabolism	Balanced lipolysis and storage	Reduced lipolysis, visceral fat gain
Insulin Sensitivity	Normal glucose uptake	Impaired, early insulin resistance
Cardiovascular Tone	Stable pressure and reflexes	Deconditioning, fluid shifts
Inflammatory Markers	Baseline cytokine levels	Elevated TNF-α and IL-6 from adipose changes

## Minimum daily movements to prevent metabolic disorders

Metabolic benefits can be achieved even at the lowest level of activity, and the risk of disorders associated with sedentary behavior can be mitigated. These intermittent motions cause muscle contractions that, in turn, promote glucose uptake and energy expenditure without the need for exercise programs (35).

In routine hospital practice, even brief periods of bed rest can lead to rapid loss of insulin sensitivity and muscle mass. These changes often occur without changes in body weight or diet and contribute to poor glycemic control and delayed recovery. This highlights the clinical importance of reducing uninterrupted mobilization whenever possible.

In spaceflight and bedrest analog studies, where voluntary movement is severely limited, muscle and metabolic decline can begin within days (36). To mitigate this effect, exercise mimetics have been used, including neuromuscular electrical stimulation (NMES), lower-body negative pressure (LBNP), and resistive vibration platforms, to simulate the mechanical and metabolic effects of gravitational loading. These activities mimic the mechanical and metabolic effects of physical exercise and have been shown to reduce muscle wasting and maintain insulin sensitivity (37, 38). In sedentary individuals or those who are bedridden, short periods of low-intensity exercise spaced throughout the day are sufficient to prevent the onset of metabolic dysfunction. This fact supports the view that even lower levels of physical activity, relative to standard exercise recommendations, are a vital daily threshold for maintaining metabolic fitness, particularly in situations characterized by forced or habitual sedentary behavior. **Table 4** summarizes the most effective low-intensity movement strategies and their associated metabolic benefits, supporting their implementation in both clinical and spaceflight contexts.

**Table 4:** Low-intensity movement strategies to prevent metabolic dysfunction.

Strategy/Modality	Mechanism	Metabolic benefit	Key references
Intermittent Low-Intensity Movement	Triggers muscle contraction without structured exercise	Improves glucose uptake and energy expenditure	(35)
NMES (Neuromuscular Electrical Stimulation)	Electrically induces muscle activity	Preserves insulin sensitivity, reduces atrophy	(37)
LBNP (Lower Body Negative Pressure)	Simulates gravity-induced blood pooling	Supports circulation, insulin regulation	(38)
Vibration Platforms	Mechanical oscillations activate postural muscles	Maintains metabolic health in sedentary states	(36, 37)

## Altered gravity’s impact on fat distribution and inflammation

When experiencing lower gravitational loading, i.e., microgravity or prolonged inactivity, the body undergoes redistribution of fat, with a tendency towards greater increases in visceral adipose tissue (VAT) (Human studies: Space flight and bed rest-data) (39). Compared to subcutaneous fat, visceral fat is highly metabolically active and plays a significant role in the development of cardiometabolic disease. Empirical studies using spaceflight and bedrest models show that accumulated visceral adipose tissue can be observed despite the absence of overall weight gain, indicating redistribution rather than an increase in adiposity (Human studies) (40).

Visceral adipose tissue (VAT) is closely associated with systemic inflammation. Visceral adipose secretes pro-inflammatory cytokines, including tumor necrosis factor-alpha (TNF-α) and interleukin-6 (IL-6), that harm the signaling pathways of insulin and worsen hepatic and vascular impairment (Human studies) (41, 42). Regular observations have shown that both astronauts and individuals in intensive care units who are immobilized exhibit elevated levels of the above cytokines, indicating an inflammatory response associated with gravitational unloading(Human studies).

The result is an immune metabolic rearrangement in which mechanical unloading triggers immune reactivity and metabolic disruption. The condition promotes the persistence of low-grade inflammation and insulin resistance, which are significant causes of obesity-related complications (43). Mechanical unloading, systemic inflammation, and insulin resistance often coexist in this setting and may worsen muscle wasting and metabolic instability, reinforcing the clinical relevance of unloading models.

Identifying the interplay among gravity, adipose tissue distribution, and inflammatory pathways is crucial for understanding the pathophysiology in sedentary individuals and in populations in spaceflight. **Table 5** summarizes these effects.

**Table 5:** Effects of microgravity and inactivity on fat redistribution, inflammation, and metabolic dysfunction, supported by empirical studies.

Aspect	Effect of microgravity/Inactivity	Key references
Fat Distribution	Shift from subcutaneous to visceral adipose tissue (VAT), despite stable weight.	(39, 40) (Human, Animal studies)
VAT Function	Metabolically active; secretes pro-inflammatory cytokines (TNF-α, IL-6)	(41, 42) (Human, *in-vitro* studies)
Inflammation	Elevated systemic cytokines observed in astronauts and bedridden individuals	(43) (Human studies)
Metabolic Consequences	Promotes insulin resistance, hepatic & vascular impairment	(41, 42) (Human, animal studies)
Pathophysiological Pattern	Immune-metabolic reprogramming triggered by unloading → chronic low-grade inflammation	(43) (Human, animal studies)

## Gravity therapy: vibration platforms and resistance suits

Without gravitational loading, the human body will rapidly deteriorate its musculoskeletal and metabolic systems. To prevent these effects, several gravity-mimicking interventions have been designed (44). Vibration platforms are among the most studied modalities and are used to stimulate muscle contraction by mechanically oscillating to simulate ground reaction forces. Empirical data demonstrate that these platforms can sustain muscle mass, bone mineral density, and circulatory activity during spaceflight and under long-duration bed rest (45).

In addition, resistive suits such as the Gravity Loading Countermeasure Skinsuit (GLCS) apply continuous axial loading across the spine and limbs. This body-weight simulation helps maintain postural muscle activity and may reduce the risk of spinal deconditioning and metabolic slowdown in microgravity environments (46, 47).

Furthermore, studies show that lower body negative pressure (LBNP) creates a vacuum around the lower limbs, drawing blood downward to simulate gravitational redistribution. This method supports cardiovascular regulation and helps maintain baroreflex function and vascular tone (48, 49).

It is the combination of these interventions that provides the necessary mechanical stimuli that gravity would otherwise provide. These modalities are thus being investigated not only among astronauts but also among patients who are not in motion or in critical condition but are living on Earth. **Table 6** outlines these major gravity-analog interventions, detailing their mechanisms and physiological benefits. Collectively, these strategies help reduce muscle atrophy, visceral adiposity, and inflammation, promoting systemic metabolic health in both microgravity and critical care settings.

**Table 6:** Gravity-mimicking interventions and their physiological benefits in counteracting microgravity-induced deconditioning.

Intervention	Mechanism of action	Targeted benefits	Key references
Vibration Platforms	Mechanical oscillations stimulate muscle contractions	Preserve muscle mass, bone density, and circulatory function	(45)
Gravity Loading Countermeasure Skinsuit (GLCS)	Applies axial loading to the spine and limbs	Supports posture, spinal health, and metabolic activity	(46, 47)
Lower Body Negative Pressure (LBNP)	Creates a vacuum around the lower limbs to draw blood downward	Maintains vascular tone, baroreflex sensitivity, and blood distribution	(48, 49)

## Role of omics data in personalized regimens

Recent developments in space omics have opened new possibilities for studying the human body’s response to microgravity at the molecular scale. Multi-omics data collection, including genomics, transcriptomics, proteomics, and metabolomics, is now routine in space missions aimed at mapping physiological adaptations to spaceflight (50, 51).

It is important to note that these molecular changes are highly individualized, depending on genetic predisposition and underlying physiological conditions. Consistent with this observation, the risks of insulin resistance, inflammation, and sarcopenia can be reduced through omics-based evaluations, enabling the development of personalized exercise, nutrition, and pharmacological approaches for astronauts (52).

This is an individualistic approach currently being applied to clinical populations on Earth. A good example is the similarity in the molecular signature of obese, diabetic people with type 2 diabetes and non-alcoholic fatty liver disease (NAFLD) and astronauts exposed to microgravity (20, 26). By incorporating omics profiling into clinical practice, it will be easier to detect people at increased risk early and to devise specific interventions that prevent or treat metabolic disorders. Therefore, space omics has not only improved the well-being of astronauts during extravehicular missions, but it also has profound implications for precision medicine on the ground. Integration of aerospace-physiology and metabolic-medicine extends the possibilities of highly-individualized therapeutic programs, especially when dealing with non-sedentary or high-risk populations with a metabolic dysfunction.

## Chart discussion: metabolic dysfunction over time and pathophysiological cascade

**Figure 1:**
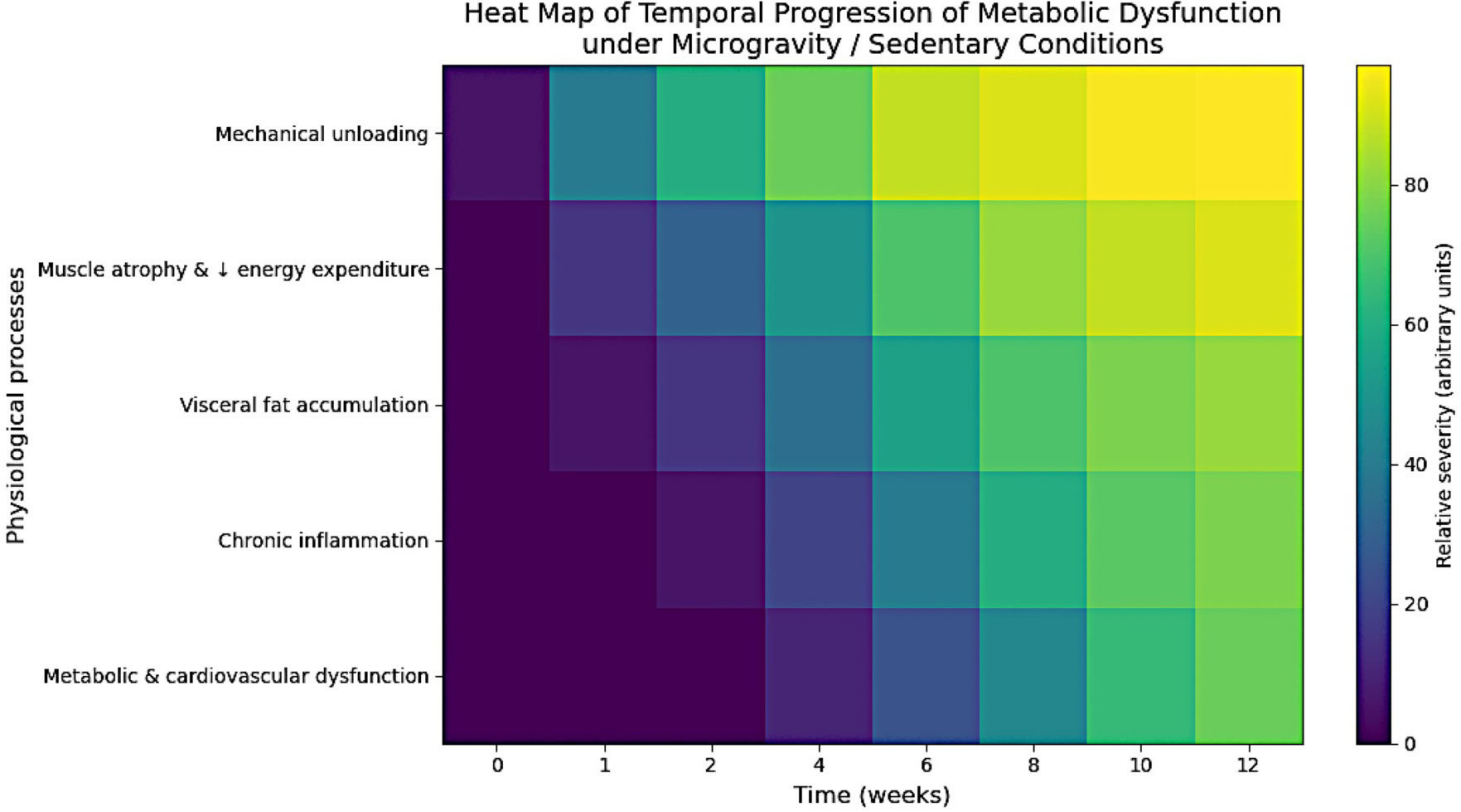
Heatmap. Physiological processes are listed on the left, and time in weeks is shown at the bottom. Initially, only mechanical unloading is observed. Over time, muscle loss occurs first, followed by fat buildup, then inflammation, and finally problems related to metabolism and the heart.

Temporal progression of physiological changes during prolonged mechanical unloading (**Figure 1**).

Pathophysiological cascade initiated by mechanical unloading, culminating in a series of inflammatory reactions. As fat accumulates, macrophage infiltration and the release of pro-inflammatory cytokines such as TNF-α and IL-6 follow, peaking between weeks 6 and 8 (53–55). This state of low-grade inflammation drives systemic metabolic stress, consistent with findings of immune dysregulation and insulin resistance in spaceflight (56).

**Figure 2:**
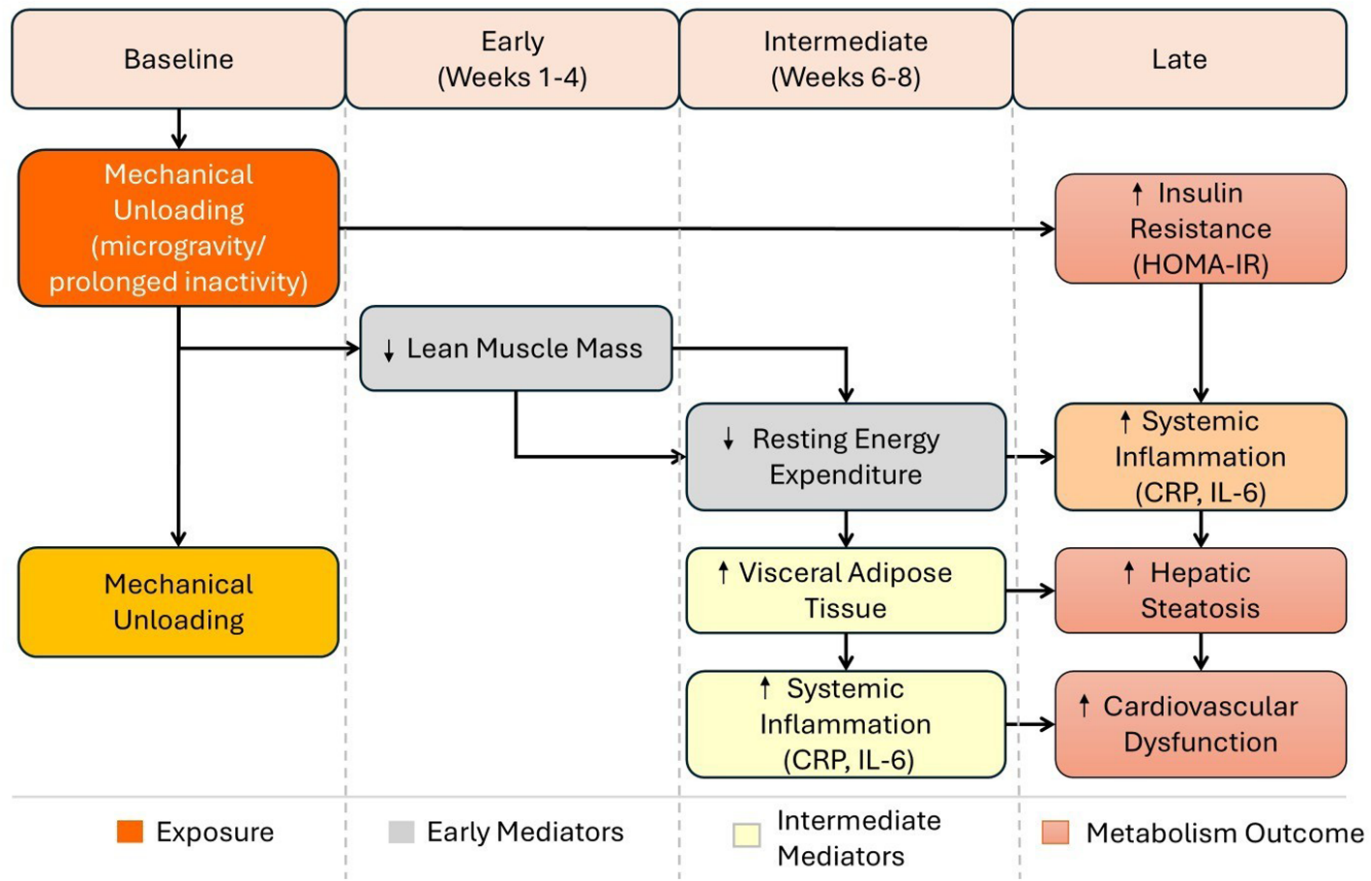
Pathophysiological cascade. The development of metabolic dysfunction over 12 weeks, either in microgravity or in a sedentary environment, reveals a sequential effect, starting with mechanical unloading and progressing to inflammatory reactions and the development of the disease.

By the twelfth week, escalation into metabolic dysfunction occurs and is characterized by hepatic steatosis and pancreatic dysfunction (57, 58). The present stage is also characterized by severe regression of cardiovascular integrity, including impaired vascular function, elevated arterial pressure, and reduced baroreflex responses. It is observed during prolonged sedentary living and in microgravity environments (59). The trends mentioned above, consistently observed in longitudinal research on prolonged bed rest and spaceflight, highlight the critical role of gravitational intactness in maintaining metabolic and immunological homeostasis (26, 27, 60–62) (**Figure 2**).

### Role of artificial intelligence in clinical translation

Artificial intelligence is increasingly being explored as a supportive tool for integrating physiological and metabolic data relevant to endocrine practice. These approaches enable earlier identification of individuals at risk of insulin resistance and ectopic fat accumulation in the liver and pancreas, thereby improving risk stratification for MASLD and MASPD. Combining biological markers across multiple domains helps explain why some individuals experience rapid muscle loss and metabolic decline, whereas others remain relatively protected, supporting more individualized prevention strategies. In routine practice, this knowledge may strengthen screening pathways, refining risk assessment, and guide follow-up in secondary or immobilized patients, improving the translation of physiological insights into everyday metabolic care (63, 64). Although artificial intelligence offers promising tools, its current role in endocrine practice is limited to supporting the establishment of diagnostic and therapeutic pathways (65, 66).

## Conclusion

This review demonstrates that microgravity and prolonged physical inactivity impair metabolic homeostasis through shared pathways, including diminished mechanical loading, altered fat distribution, inflammation, and insulin resistance. Spaceflight and sedentary lifestyles share many similarities; therefore, microgravity is a valuable tool for understanding obesity, type 2 diabetes, and ectopic fat deposition in the liver and pancreas. Older adults with lower daily movement, combined with age-related muscle loss, can promote insulin resistance and visceral fat accumulation, contributing to sarcopenic obesity and metabolic decline. Although gravity-mimicking interventions and omics-based personalized regimens are promising, their effectiveness across populations remains untested. Many results are based on short-duration studies or extrapolations from animal models, making generalization difficult. Subsequent studies ought to be based on longitudinal, multi-omics human studies across a variety of populations and non-experimental sedentary conditions. The further opening of spaceflight data and its integration with Earth-based clinical trials could mark the transition from aerospace physiology to metabolic medicine and eventually enable individualized dosing for lifestyle-related diseases. Further studies integrating mechanical loading assessment and metabolic phenotyping may establish mechanical unloading as a measurable and modifiable target in endocrine risk stratification.
